# O03 A case of hyperinflammatory COVID-19 that responded to tocilizumab therapy

**DOI:** 10.1093/rap/rkaa053.002

**Published:** 2020-11-03

**Authors:** Kathryn Biddle, Daniel Burrage, Nidhi Sofat

**Affiliations:** St George’s University Hospitals NHS Foundation Trust, London, United Kingdom

## Abstract

**Case report - Introduction:**

Coronavirus disease 19 (COVID-19), caused by Severe Acute Respiratory Syndrome Coronavirus (SARS-CoV-2), has reached pandemic level and led to over 46,000 deaths in the UK. COVID-19 is primarily a respiratory illness and 10-20% of infected individuals develop severe disease with interstitial pneumonia or acute respiratory distress syndrome (ARDS). In this subgroup of patients, severe clinical manifestations are postulated to result from a hyperactive immune response. This has led to the proposal that immunomodulatory medications could be used for the treatment of COVID-19. Here, we report a case of COVID-19 that was treated with the IL-6 inhibitor, tocilizumab.

**Case report - Case description:**

A 54-year-old Middle Eastern woman presented to A&E with a one-week history of fever, cough, headache and ageusia. Her past medical history was significant for asthma, chronic headaches, gastro-oesophageal reflux syndrome and subarachnoid haemorrhage. On presentation, she had a low-grade temperature (37.8 °C) but her observations were otherwise normal, and her oxygen saturations were 99% on room air. Examination revealed right basal chest crackles. Bloods showed a mild lymphopenia (0.9x10^9^/l) and a raised CRP (82mg/l) and a chest radiograph demonstrated bibasal shadowing. The patient was diagnosed with probable COVID-19 and discharged with a course of oral doxycycline and a plan for review in the ambulatory unit the following day. When reviewed the next day, her oxygen saturations had fallen to 90% on room air. At this point, her SARS-CoV-2 assay had been resulted as positive and a decision was made to admit her for oxygen therapy.

The patient continued to deteriorate despite optimal supportive therapy and the addition of intravenous benzylpenicillin for possible superadded bacterial infection. On day 7 of admission, her respiratory rate was 32-38 breaths per minute, and she required 13l/min of oxygen. Her bloods revealed CRP 474mg/L, D dimer >6000 ng/ml, ferritin 224 μg/L, neutrophils 9.5x10^9^/l and lymphocytes 0.6 x10^9^/l. There were no signs of superadded bacterial infection despite a thorough infection screen. Given her clinical deterioration, she was reviewed by the critical care team for consideration of transfer to higher-level care. The ward team decided to administer a single dose of the anti-IL-6 agent tocilizumab for the treatment of a cytokine storm secondary to COVID-19 infection.

Within 24 hours of tocilizumab treatment, her oxygen requirements fell to 5l/min and her work of breathing significantly improved. On day 15 of admission, she was discharged with saturations of 92% on room air.

**Case report - Discussion:**

The patient described in this case showed significant clinical deterioration with features suggestive of cytokine storm secondary to COVID-19. IL-6 is thought to be a key cytokine responsible for initiating the acute phase response and we postulate that IL-6 levels were raised in this patient. Unfortunately, we did not have the assay available to measure this. The treating clinical team decided to prescribe a single dose of tocilizumab on a compassionate use basis. This resulted in a rapid clinical improvement and the patient was subsequently discharged without the need for intensive care. In this case, we propose that tocilizumab inhibited further cytokine activation and prevented the positive feedback loop of inflammation that can otherwise result in rapid clinical deterioration.

There are several interesting points to be noted from this case. In this patient, tocilizumab resulted in a rapid reduction in CRP levels. This is thought to correspond to the inhibition of IL-6 mediated release of acute phase proteins by the liver. Therefore, it should be noted that post-tocilizumab treatment, patients should be closely monitored for superadded bacterial infection as they may not mount a full immune response.

Larger trials of tocilizumab for the treatment of COVID-19 are currently underway and are required to confirm the efficacy of IL-6 inhibition for COVID-19. The phase III COVACTA trial of tocilizumab in COVID-19 patients did not meet its primary endpoint of improved clinical status however a trend towards shorter hospital admissions was seen. Further studies are ongoing to investigate the role of tocilizumab in other treatment settings, including in combination with an antiviral medication. Further information is required to determine which patients should receive immunomodulatory medications and at which point in their illness. Data is also needed to understand the most efficacious dosing regimen for tocilizumab and its side-effect profile in COVID-19 patients.

**Case report - Key learning points:**

The COVID-19 pandemic has affected millions of people worldwide and has led to an unprecedented effort from the scientific community to understand the pathophysiology of the disease and to find effective treatments. Emerging evidence suggests that SARS-CoV-2 can induce a hyperactive immune response in a subgroup of patients who develop highly elevated levels of acute phase proteins. It has been proposed that the overactive immune response is responsible for some of the severe clinical manifestations seen and this has led to the suggestion that immunomodulatory medications could be used for the treatment of COVID-19.

Indeed, dexamethasone has been shown to be an effective treatment and other immunomodulatory medications including hydroxychloroquine, the IL-1 inhibitor anakinra and JAK-kinase inhibitors are currently being trialled for the treatment of COVID-19. This case highlights the clinical and biochemical features of a patient who developed features suggestive of a cytokine storm secondary to COVID-19 and who responded to treatment with the IL-6 inhibitor tocilizumab. Further work is required to understand the role of immunomodulatory medications for the management of COVID-19 infection.

